# Spatiotemporal Expression of p63 in Mouse Epidermal Commitment

**DOI:** 10.3390/ijms161226185

**Published:** 2015-12-10

**Authors:** Qian Zhao, Shuang Liu, Huishan Zhang, Na Li, Xinyue Wang, Yujing Cao, Lina Ning, Enkui Duan, Guoliang Xia

**Affiliations:** 1 State Key Laboratory of Agrobiotechnology, College of Biological Sciences, China Agricultural University, Beijing 100193, China; bingxia0923@163.com; 2 State Key Laboratory of Stem Cell and Reproductive Biology, Institute of Zoology, Chinese Academy of Sciences, Beijing 100101, China; liushuang@ioz.ac.cn (S.L.); zhanghs@ioz.ac.cn (H.Z.); linawhere@163.com (N.L.); xywang@ioz.ac.cn (X.W.); caoyj@ioz.ac.cn (Y.C.); ningln@ioz.ac.cn (L.N.); 3 University of Chinese Academy of Sciences, Beijing 100049, China

**Keywords:** p63, spatiotemporal expression pattern, K8, K5/K14, epidermal commitment

## Abstract

The embryonic surface ectoderm is a simple flat epithelium consisting of cells that express the cytokeratins K8/K18. Before stratification, K5/K14 expression substitutes K8/K18 expression, marking the event called epidermal commitment. Previous studies show that the transcription factor p63 plays an essential role in epidermal commitment. However, detailed expression information of p63 during early epidermal development in mice is still unclear. We systematically studied the expression pattern of p63 in mouse epidermal commitment, together with K8 and K5. We show that p63 expression could be detected as early as E8.5 in mouse embryos preceding epidermal commitment. p63 expression first appears near the newly formed somites and the posterior part of the embryo, further expanding to the whole embryonic surface with particular enrichment in the first branchial arches and the limb buds. ΔNp63 is the major class of isoforms expressed in this period. Relative expression intensity of p63 depends on the embryonic position. In summary, there is a sequential and regular expression pattern of K8, p63 and K5 in mouse epidermal commitment. Our study not only contributes to understanding the early events during epidermal development but also provides a basal tool to study the function of p63 in mammals.

## 1. Introduction

The mammalian epidermis is a multi-layered epithelium containing some elaborate appendages to protect the organism against external insults [1]. During embryonic development, the epidermis derives from the single-layered surface ectoderm, which expresses the paired keratins, Keratin 8 (K8) and Keratin 18 (K18), and generates a transient protective layer called periderm, which is shed off once the epidermis has differentiated and stratified [2,3,4]. Before stratification, around E9.5 in mouse embryos, expression of the keratin pair Keratin 5/Keratin 14 (K5/K14) starts to replace K8/K18 expression, which marks the developmental event called the epidermal commitment [5]. All the structures of the future epidermis originate from this embryonic epidermal basal layer, which expresses K5/K14 [6]. In the wake of epidermal commitment, the epidermis initiates stratification. The proliferating cells from the basal layer migrate upward and mature into the spinous and granular layers [7,8]. The process of terminal differentiation is finished by forming the cornfield layer which establishes the function of the skin barrier [9]. After birth, the epidermal homeostasis and self-renewal rely on the proliferation and differentiation of basal layer cells. Since the stratification of the epidermis and the formation of various accessory structures are all based on the basal layer, the epidermal commitment process, in which the epidermal basal layer is established, is of elementary importance for the entire morphogenesis and maturation of the skin epidermis. Outstanding works in recent years have shed light on skin epidermal stratification and differentiation, but the early events regarding the epidermal commitment are ill stated.

p63 is a member of the p53 tumor suppressor gene family and contains two major classes of isoforms, TAp63 and ΔNp63. p63 is known as a master transcription factor in epithelial development and differentiation. In particular, it is required for the maintenance of proliferation potential in epithelial stem cells [10,11], the commitment of the epithelial lineage [12,13], the differentiation of keratinocytes [14,15] and the adhesion and survival of epithelial cells [16,17,18]. Abnormal expression of p63 is closely related to ectodermal dysplastic syndromes and cancer development in humans [19]. The critical role of p63 in mouse epidermal development is demonstrated by the phenotype of p63 knockout (p63^−/−^) mice, which failed to form the stratified epidermis, limbs, mammary gland and tooth, resulting in early postnatal lethality due to severe dehydration [11,20]. Surface epithelial cells in p63^−/−^ mouse express K18, the marker for single-layered epithelia, while K5/K14 expression or other epidermal differentiation markers could not be detected, indicating the developmental block during the epidermal commitment process [15,20]. Supporting that, p63 directly or indirectly governs the expression of K14 during this process [21,22,23]. Although several studies have identified the roles and molecular mechanisms of p63 during subsequent epidermal development, when and where p63 is expressed and how it functions during epidermal commitment is largely unknown.

In the present study, we systematically investigated the expression pattern of p63 around the period of mouse epidermal commitment (from E8.5 to E10.5), together with K8 and K5/K14 expression. We revealed an interesting expression mode of p63 during this process and discovered sequential and interesting expression patterns of these genes. Our study contributes to understanding the early events during epidermal development and offers a basal tool to study the function of the transcription factor p63 in mammals.

## 2. Results and Discussion

### 2.1. The Spatiotemporal Expression of Markers Related to Epidermal Commitment

**Figure 1:**
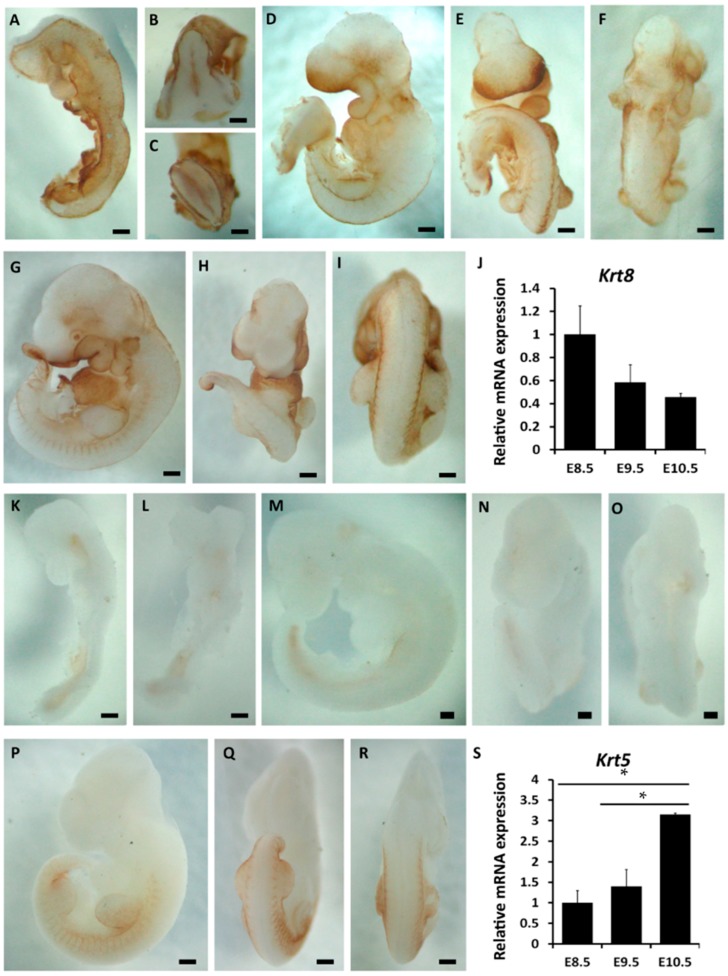
The spatiotemporal expression of markers related to epidermal commitment from E8.5 to E10.5 by whole-mount immunohistochemistry. (**A**–**I**) Whole-mount immunostaining of K8 in mouse embryos at E8.5 ((**A**) lateral; (**B**) cephalic; (**C**) caudal view), E9.5 ((**D**) lateral; (**E**) ventral; (**F**) dorsal view) and E10.5 ((**G**) lateral; (**H**) ventral; (**I**) dorsal view); (**J**) qRT-PCR result showing *Krt8* mRNA expression at different embryonic days during epidermal commitment; (**K**–**R**) Whole-mount immunostaining of K5 in mouse embryos at E8.5 ((**K**) lateral; (**L**) ventral side), E9.5 ((**M**) lateral; (**N**) ventral; (**O**) dorsal view), E10.5 ((**P**) lateral; (**Q**) ventral; (**R**) dorsal view); (**S**) qRT-PCR result showing *Krt5* mRNA expression at different embryonic days during epidermal commitment. Bar = 200 μm for (**A**–**F**) and (**K**–**O**); bar = 500 μm for **G**–**I** and (**P**–**R**). * *p* < 0.05.

During epidermal commitment, it is a hallmark that K5/K14 expression substitutes K8/K18 expression. To understand more about the process of epidermal commitment, we first investigated the expression patterns of K8 and K5 from E8.5 to E10.5. As indicated by whole-mount immunohistochemical staining, at E8.5, K8 was strongly expressed in the surface ectoderm which was a monolayer epithelium (Figure 1A–C and Figure S1A,B). The negative controls for whole-mount immunohistochemistry were provided in Figure S2. However, it was absent in neuroectodermal cells (Figure S1A). At E9.5, the expression of K8 still covered the whole embryo, but the expression intensity in the dorsal half of the embryo was lower than that in the ventral half (Figure 1D–F). Subsequently at E10.5, it decreased in the entire embryonic surface, especially in the surface ectoderm covering the somites and the limb buds (Figure 1G–I). Further we employed qRT-PCR to examine the relative expression level of K8 at different time points. Since the proportion of the surface ectodermal cells in the embryo was very small, it was difficult to isolate pure surface ectodermal cells. Therefore, the entire embryo was used instead of the pure surface ectoderm in qRT-PCR to evaluate the general change in the expression levels of *Krt8* and *Krt5* which were mainly expressed in the surface ectoderm during this period. The results of qRT-PCR indicated that there was a downward trend of *Krt8* mRNA expression of in surface ectoderm from E8.5 to E10.5 (Figure 1J).

Through whole-mount immunohistochemistry, it was observed that K5 expression in the mouse embryos could be first detected in the limb buds surface at E9.5 and then it started to expand to the posterior somatic surface at E10.5 (Figure 1K–R). That was more clearly shown by the sections of whole-mount-immunostained embryos (Figure S1C–G). At the same time, qRT-PCR results also testified that the expression of *Krt5* increased from E8.5 to E10.5 (Figure 1S). Summing the results, K8 expression was abundant in the surface, whereas K5 expression was not turned on at E8.5. One day later, K8 expression still covered the surface and K5 was detected only in very few regions. At E10.5, K8 expression diminished in some locations, whereas K5 expression became robust in certain regions.

### 2.2. The Spatiotemporal Expression of p63 in Mouse Embryos from E8.5 to E10.5

**Figure 2:**
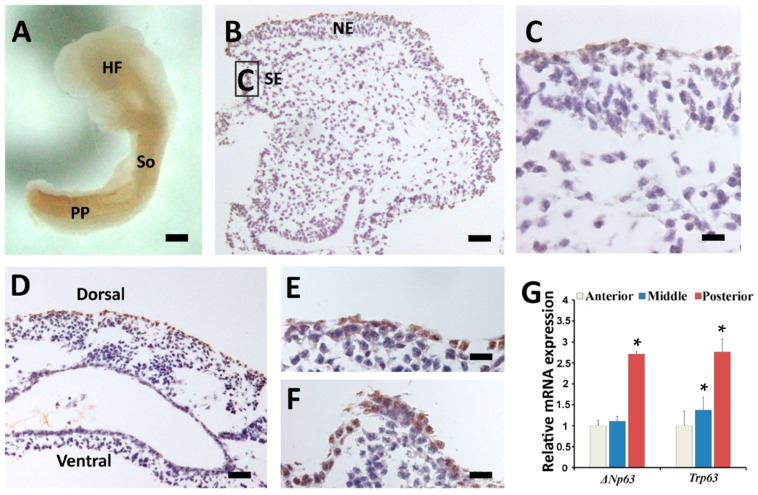
p63 protein and mRNA expression in E8.5 mouse embryos. (**A**) Lateral view of an E8.5 embryo stained with p63 antibody; (**B**–**F**) Lateral sections showing different parts of the p63-stained E8.5 embryo. (**B**,**C**) Head, region that is magnified in **C** is boxed in **B**; (**D**) Trunk region containing the somites; (**E**) A larger view of the surface ectoderm covering the somite; (**F**) Surface ectoderm and the underlying mesenchyme of the posterior part of the embryo; (**G**) qRT-PCR results showing the mRNA expression of Δ*Np63* and *Trp63* in anterior, middle, and posterior parts of the embryo. HF, head field; So, somite; PP, posterior part of the embryo; Ne, neuroectoderm; SE, surface ectoderm. Bar = 200 μm for (**A**); bar = 50 μm for (**B**) and (**D**); bar = 20 μm for (**C**), (**E**) and (**F**). * *p* < 0.05 when compared with 1.

p63 was faintly observed outside the newly formed somites and the posterior part of the embryo preceding epidermal commitment at E8.5 (Figure 2A). In order to clearly demonstrate the expression of p63 at this time point, the stained embryo was sectioned. Interestingly, p63 was undetectable in the head field ectoderm (Figure 2B,C), but it was intensively expressed in the ectoderm overlying the dorsal somites and the posterior portion of embryo (Figure 2D–F). Furthermore, we used qRT-PCR to evaluate the region-specific relative expression of *TAp63*, *ΔNp63* and *Trp63* mRNA in E8.5 embryo. The results showed that mRNA expression patterns of *ΔNp63* and *Trp63* were similar (Figure 2G), but *TAp63* expression was too low to be accurately evaluated (the CT values were too high, meaning very little expression). Therefore, the spatiotemporal expression of p63 actually represented the specific expression of ΔNp63. Consistent with the whole-mount immunostaining, *Trp63*/*ΔNp63* mRNA expression was higher in the somites and the posterior portions than that in the head portion (Figure 2G). These results indicated that p63 expression could be detected as early as E8.5 in mice preceding epidermal commitment and it first appeared near the newly formed somites and the posterior part of embryo.

**Figure 3:**
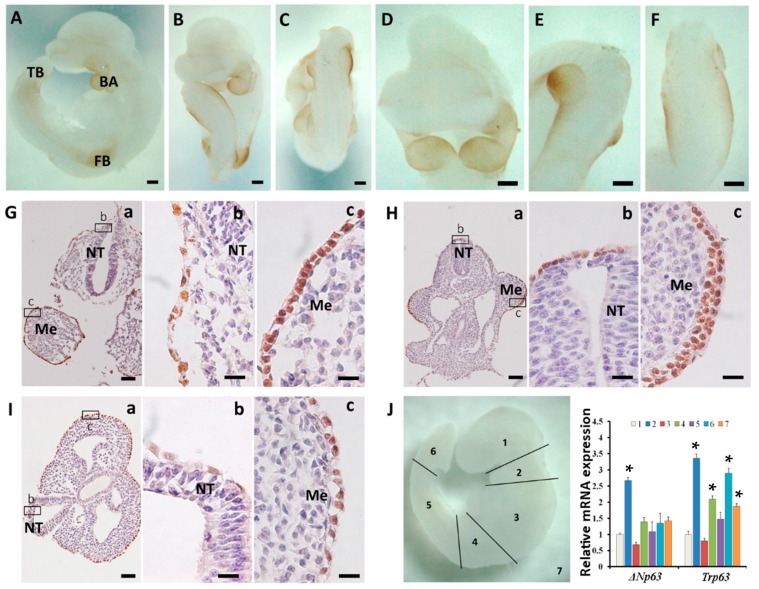
p63 expression in E9.5 embryos. (**A**–**C**) Different views showing the whole-mount immunostaining of p63 ((**A**) lateral; (**B**) ventral; (**C**) dorsal view). (**D**–**F**) Images with higher magnification showing the head (**D**), forelimb buds (**E**) and the tail bud (**F**). (**G**–**I**) Transverse sections of the whole-mount stained E9.5 embryo. (**B**,**C**) show the boxed areas in (**A**), (**G**) head; (**H**) forelimb buds; (**I**) tail bud; (**J**) qRT-PCR results showing relative *ΔNp63* and *Trp63* mRNA expression in different regions of the E9.5 embryo. Note that the numbers in the **right** panel of **J** are corresponded to the numbers in the **left** panel of **J**. BA, branchial arch; FB, forelimb bud; TB, tail bud; NT, neural tube; Me, mesenchyme. Bar = 200 μm for (**A**–**F**); bar = 50 μm for **Ga**, **Ha** and **Ia**; bar = 20 μm for **Gb**,**c**, **Hb**,**c** and **Ib**,**c**. * *p* < 0.05 when compared with 1.

At E9.5, it was obviously noted that p63 expression was intense in the surface ectoderm covering the first branchial arches, the forelimb buds and the tail bud, whereas other regions only showed faint staining (Figure 3A–C). This regional pattern was more evident with larger magnification of specific sites (Figure 3D–F). Sections of E9.5 mouse embryos confirmed that p63 was strongly expressed in the first branchial arches, the forelimb buds and the tail bud (Figure 3G–I). Sections of the head showed that the expression of p63 in the surface ectoderm close to the mesenchyme was more intensive than that close to the neural tube (Figure 3G). Sections of the forelimb buds indicated that p63 violently appeared in the apical ectodermal ridge (ARE) of the limb, which was closely connected to the mesenchyme (Figure 3H). Sections of the tail bud also exhibited a similar p63 expression pattern to that of the head sections, *i.e.*, the surface epidermal cells close to mesenchyme showed more intensive p63 expression than that close to neural tube (Figure 3I). In addition, qRT-PCR results showed that the mRNA expression of *Trp63* in the first branchial arches (2), the forelimb buds (4), and the tail bud (6) were significantly higher compared with that of the head (1) (Figure 3J). The expression pattern of *ΔNp63* mRNA was generally similar to *Trp63*, but *TAp63* mRNA expression was hardly detected (Figure 3J). This evidence revealed that p63 expression exhibited a specific regional pattern and ΔNp63 was the major class of isoforms expressed in the E9.5 embryo.

**Figure 4:**
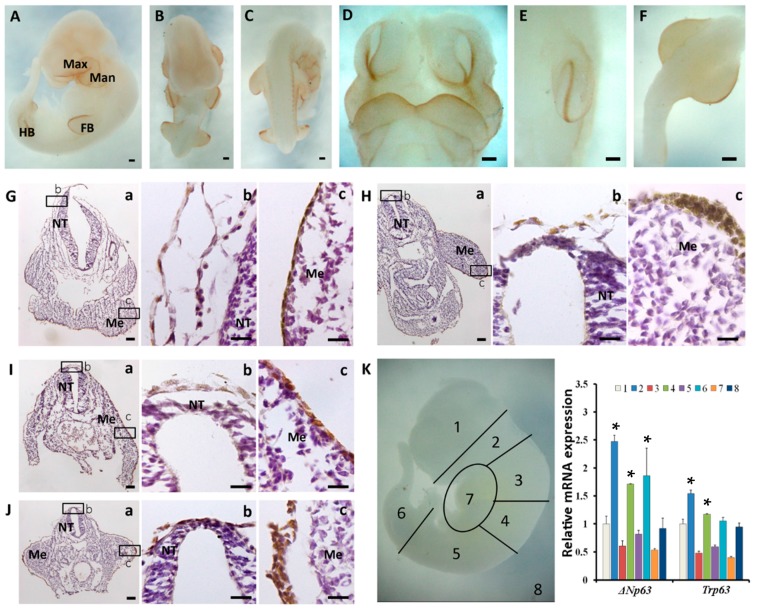
p63 expression in E10.5 embryos. (**A**–**C**) Different views showing the whole-mount immunostaining of p63 (**A**) lateral; (**B**) ventral; (**C**) dorsal view); (**D**–**F**) Images with higher magnification showing the head (**D**); the forelimb bud (**E**) and the hindlimb buds (**F**); (**G**–**J**) Transverse sections of the whole-mount-stained E10.5 embryo. b and c show the boxed areas in a, (**G**) head; (**H**) forelimb buds; (**I**) somite; (**J**) hindlimb buds; (**K**) qRT-PCR results showing relative *ΔNp63* and *Trp63* mRNA expression in different regions of the E10.5 embryo. Note that the numbers in the **right** panel of **K** are corresponded to the numbers in the **left** panel of **K**. Max, maxillary; Man, mandibular; FB, forelimb bud; HB, hindlimb bud; NT, neural tube; Me, mesenchyme. Bar = 200 μm for (**A**–**F**); bar = 100 μm for **Ga**, **Ha**, **Ia** and **Ga**; bar = 20 μm for **Gb**,**c**, **Hb**,**c**, **Ib**,**c** and **Jb**,**c**. * *p* < 0.05 when compared with 1.

At E10.5, the expression of p63 expanded to the whole embryonic surface, and it was more evident in the maxillary and mandibular regions of the arch epithelium, forelimb buds and hindlimb buds (Figure 4A–F). E10.5 sections showed a comparable p63 expression pattern with E9.5 sections, that was, the mesenchyme-related surface ectoderm showed higher intensity of p63 expression than the neural tube-related surface ectoderm (Figure 4G–J). The results of qRT-PCR also indicated that the expression of *Trp63* mRNA in the first branchial arches (2) and the forelimb buds (4) was higher than that in the head (1), which was also the case for *ΔNp63* mRNA expression (Figure 4K). *TAp63* mRNA expression was still hardly detected. These data further proved that p63 expression depended on the position of the embryo and ΔNp63 was the major class of isoforms expressed in the E10.5 embryo.

To compare the p63 expression from E8.5 to E10.5, we conducted qRT-PCR to evaluate the relative *Trp63* mRNA expression level in the entire embryo. The results showed that *Trp63* mRNA expression increased with time (Figure S3). In summary, expression of p63 was detected as early as E8.5. It was expressed in the surface ectoderm covering the newly formed somites and the posterior part of embryo preceding epidermal commitment. Subsequently, it was detectable in the surface ectoderm overlying the branchial arches and forelimb buds and the tail bud regions at E9.5. At E10.5, the expression was evident in the maxillary and mandibular regions of the arch epithelium and the limb buds, and then expanded to the whole embryo. It was observed that the surface ectoderm close to the mesenchyme showed stronger p63 expression than that close to the neural tube. ΔNp63 is the predominant class of isoforms expressed at E8.5–E10.5 whereas TAp63 expression was hardly detected. Therefore, the spatiotemporal expression of universal p63 largely represented the specific expression of ΔNp63. Taken together, the expression of p63/ΔNp63 exhibited a highly region-specific pattern.

### 2.3. Sequential Expression of K8, p63 and K14 during Mouse Epidermal Commitment

**Figure 5:**
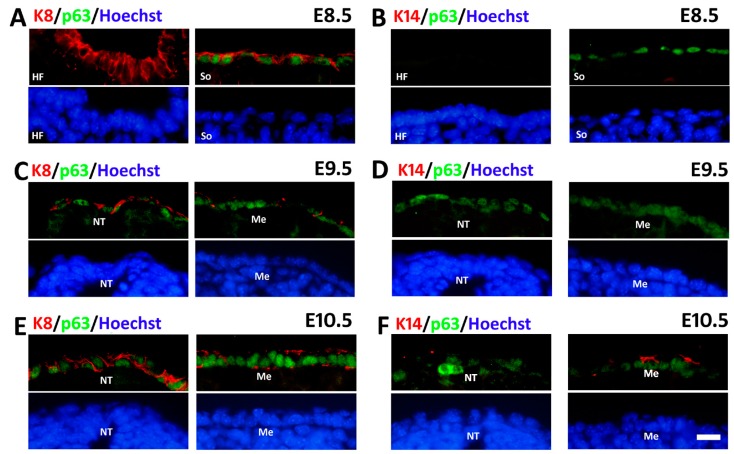
Double immunofluorescence staining of p63 with K8 and K14, respectively. (**A**,**C**,**E**) Immunofluorescense analysis of K8 (red) and p63 (green) during mouse skin development at E8.5–E10.5 (**B**,**D**,**F**) Immunofluorescense analysis of p63 (green) and K14 (red), during mouse skin development at E8.5–E10.5 Hoechst33342 was used to indicate the nuclei. HF, head field; So, somite; NT, neural tube; Me, mesenchyme. Bar = 20 μm.

In order to examine the relationship between p63 and the epidermal commitment-related markers, we performed double staining of p63 together with K8 or K14, the partner keratin of K5 in the mouse embryo. At E8.5, p63 was expressed in the surface of somites (Figure 5A, right) rather than in the head field surface (Figure 5A, left) whereas K8 expression was abundant at both sites. Moreover, K14 expression was detected in neither region (Figure 5B). At E9.5, K8 expression was still robust in the surface covering the neural tube (Figure 5C, left) but diminished in the surface covering the mesenchyme (Figure 5C, right). In the meantime, p63 expression emerged overlying the neural tube (Figure 5C, left) while it became more abundant outside the mesenchyme (Figure 5C, right). However, both analyzed regions hardly showed any K14 expression yet (Figure 5D) although it was detected in the limb bud ectoderm as aforementioned. At E10.5, K8 and p63 expression exhibited a similar expression pattern with E9.5 that K8 expression in the surface ectoderm was stronger close to the neural tube (Figure 5E, left) than that close to the mesenchyme (Figure 5F, right). At the same time, p63 expression was more intensive over the mesenchyme (Figure 5E, right) than neural tube (Figure 5F, left). K14 expression only appeared near the mesenchyme where p63 expression was intensive (Figure 5F). The data above enunciated that p63 expression was initiated after K8 and before K14 during ectoderm development and the epidermal commitment first happened in some specific areas where p63 expression was concentrated.

## 3. Discussion

There are two major classes of p63 isoforms, TAp63 and ΔNp63. ΔNp63 isoforms lack the N-terminal transactivating domain [24]. The Koster group reported that TAp63 was detected as early as E7.5 prior to the commitment to stratification and was convinced that TAp63 was the main isoform expressed in early epidermal morphogenesis [15,21]. We found that *DNp63* mRNA was abundant at E8.5–E10.5 whereas *TAp63* mRNA was only faintly detected at E10.5 (Figure 2G, Figure 3J, Figure 4K and Figure S4A). Besides, we conducted immunohistochemistry to detect the specific expression of DNp63 isoforms and discovered that ΔNp63 expression was clearly seen in surface ectoderm cells of the newly formed somites and the lateral plate mesenchyme at E8.5 (Figure S4B) which was indistinguishable from the results we obtained with the antibody against all p63 isoforms. These data indicate that ΔNp63 isoforms are the major class expressed during this period and the expression pattern of p63 largely represents the expression of ΔNp63, consistent with some other groups who hold that ΔNp63 plays the critical role in early epidermal morphogenesis [23,25,26].

Previously, one study has mentioned that *Trp63* mRNA expression could be detected in E8 embryos, but the details were not clearly stated [25]. We provided detailed information about the p63 expression in the E8.5 mouse embryos, especially in the dorsal surface ectoderm where it covers the paraxial mesoderm. The data about E9.5 and E10.5 are in accordance with the previous reports in which the *Trp63* mRNA expression was investigated by *in situ* hybridization [11,20]. We further discovered that the expression of p63 in surface ectoderm near the mesenchyme is more intensive than that close to the neural tube. During early embryonic development, organogenesis is directed by reciprocal and sequential interactions between the epithelium and the mesenchyme [27]. The recombination of epithelium and mesenchyme has shown that the pattern and shape of organs appear to be regulated by the mesenchyme, which indicates that the mesenchyme seems to supply the first signals directing organogenesis in many organ studies, such as the formation of feather follicles in chicken, the morphogenesis of tooth and the development of the mammary gland [5,28]. In hair and feather development, Wnt family molecules might be involved in the first mesenchymal message [29]. Besides, signaling molecules of the Shh, FGF, BMP and Wnt families appear to regulate the early steps of tooth morphogenesis [30]. The surface ectoderm is a single-layered epithelium originating from the lateral portions of the ectoderm and differentiation to the skin epithelium, the major part of hair follicles and some ectodermal organs. According to these region-specific data, we speculate that there might be some extracellular signals derived from the mesenchyme that initiates p63 expression to regulate the process of epidermal commitment.

Additionally, we investigated the sequential expression patterns of K8, p63 and K14 from E8.5 to E10.5. We find that p63 expression is initiated after K8 expression and before K14 expression and that K14 expression first appears where p63 expression is intensive. Recently, it has been reported that ΔNp63 played an important role in directly regulating the expression of K5 and K14 through binding to the enhancer and prompter elements in keratinocyte cell lines *in vitro* [22,31,32]*.* Candi *et al.* have determined that ΔNp63 directly regulated K14 expression after being recruited to the minimal promoter region of *Krt14* in inducible cell lines [23]. In addition, microarray data also illustrate that *Krt14* was one of the target genes of p63 [14,33,34]. On the other hand, the data of the Koster group demonstrated that TAp63 could indirectly regulate *Krt14* gene expression by upregulating AP-2γ in epidermal morphogenesis [21]. We believe that any functional study of p63 should be based on a clear demonstration of when and where they are expressed. In view of our data that ΔNp63 is the major class of isoforms that is expressed at E8.5–E10.5, we don’t agree with the role of TAp63 in early epidermal morphogenesis. The regulatory mechanism of p63 governing keratin genes expression may be more complex in the skin epidermis *in vivo* as compared to that in cultured cells. Interestingly, the expression of K5/K14 does not necessarily correlate with p63 expression in the entire surface. For instance, K5 is expressed following p63 expression in the limb bud, but it is not turned on in the branchial arches even though p63 expression is intensive. We assume that other signals, possibly derived from the underlying mesenchyme, might also be required to initiate K5/K14 expression. The mesenchymal cells that contribute to the limb bud are derived from the axial mesoderm and the mesenchymal cells of the branchial arches are originated from cranial neural crest cells [35]. It is possible that these different mesenchymal cells produce distinct signals, some of which might be required for K5/K14 expression. The nature of signals that mediates and/or coordinates with p63 during this process is still ill-defined. It would be interesting to use new techniques and methods to uncover these signals in epidermal commitment.

## 4. Experimental Section

### 4.1. Animal and Embryo Preparation

All mouse experiments were handled according to animal care protocols approved by the Guideline of the Ethics Committee of the Institute of Zoology, Chinese Academy of Science (CAS) and supported by the funding of the Strategic Priority Research Program of the CAS (XDA01010202, from 1 January 2011 to 31 December 2015). CD1 mice were purchased from Vital River Laboratory Animal Center, Beijing, China. Adult CD1 mice were mated and the day of the appearance of the vaginal plug was taken as E0.5. The pregnant mice were sacrificed and the embryos were collected on the day of analysis.

### 4.2. Whole-Mount Immunohistochemistry of Mouse Embryos

Immunohistochemistry of whole-mount mouse embryos were performed as previously described [36]. Briefly, microdissected embryos were fixed in methanol/DMSO (all from Sigma, St. Louis, MO, USA) (4:1) at 4 °C overnight and then transferred into methanol/DMSO/H_2_O_2_ (all from Sigma) (4:1:1) at room temperature for 5–10 h to block endogenous peroxidase activity. Samples were rehydrated in gradient methanol successively and blocked in blocking buffer-PBSMT (2% nonfat instant skim milk, 0.5% Triton X-100 in phosphate buffered saline-PBS) (all from Sigma) for 1 h at room temperature. The embryos were incubated with specific dilution of primary antibodies in PBSMT overnight at 4 °C with rocking, then rinsed in PBSMT three times. The embryos were incubated with corresponding secondary antibodies (Zhongshan Goldenbridge Biotechnology Co., Ltd., Beijing, China) also diluted in PBSMT (1:500) at 4 °C overnight. The embryos were subsequently washed in PBSMT before added with diaminobenzidine (DAB) (Zhongshan Goldenbridge Biotechnology Co., Ltd.). 0.03% H_2_O_2_ was successively added when the samples looked good. Then, embryos were photographed on 15% agarose gel (Sigma) to maintain the desired positions. 

The primary antibodies are listed in Table S1. The data showing specific staining using these antibodies were provided in Figure S5 as a visual reference.

The embryos were washed in PBS and then dehydrated in 30% sugar (Sigma) at 4 °C overnight. The embryos were then embedded in optimal cutting temperature compound (Sakura Finetek Co., Ltd., Torrance, CA, USA), frozen, and cryosectioned into 10 μm-thick slices using the CM1950 platform (Leica Instruments, Nussloch, Germany). Slides were stained with hematoxylin (Zhongshan Goldenbridge Biotechnology Co., Ltd.) for nuclear identification and subsequently dehydrated in gradient alcohol, mounted with neutral resin and then examined and photographed under a Nikon Fluorescence Microscope E80i (Nikon, Tokyo, Japan).

### 4.3. Reverse-Transcription and Polymerase Chain Reaction (qRT-PCR and RT-PCR)

RNA was extracted and purified from each sample with ReliaPrep™ RNA Cell Miniprep System (Promega, Madison, WI, USA). cDNA was synthesized using GoScript™ Reverse Transcription System (Promega) according to the manufacturer’s protocol. 

qPCR was conducted using GoTaq^®^ qPCR Master Mix (SYBR Green) (Promega) on a Roche LightCycler480 system (Roche Applied Science, Indianapolis, IN, USA) as previously described [37,38]. Primers used in these experiments are listed in Table S2. The qPCR procedure was as follows: 94 °C for 2 min followed by 40 cycles of 95 °C for 15 s, 55 °C for 15 s and 68 °C for 25 s. The relative mRNA expression of each gene normalized to *Gapdh* expression was calculated using the −ΔΔ*C*_t_ method with efficiency correlation [39]. More than three independent samples were used for qPCR assays, and the differences between two groups were analyzed with the independent sample *t*-test. Data were presented as mean ± SEM. *p* value < 0.05 was considered statistically significant.

After the generation of cDNA, PCR was performed using Promega PCR Master Mix (Promega) as described previously [40]. The PCR procedure was as follows: 95 °C for 5 min; 25 or 30 cycles of 95 °C for 30 s, 56 °C for 30 s and 72 °C for 30 s and 72 °C for 5 min. The primers used to amplify different genes are also listed in Table S2. *Gapdh* was used as an internal control.

### 4.4. Immunofluorescence Double Staining and DAB Immunohistochemistry

Microdissected mouse embryos were fixed in 4% paraformaldehyde (PFA) (Sigma) in PBS at 4 °C overnight and transferred embryos into 30% sugar (Sigma) to dehydrate overnight, and then embedded and frozen in optimal cutting temperature compound (Sakura Finetek Co., Ltd.) 10 µm-thick embryonic frozen sections were washed in PBS for three times and boiled in the sodium citrate Buffer (0.1 mol citric acid monohydrate and 0.1 mol citric acid dehydrate in 1000 mL H_2_O) (Sigma) for antigen retrieval. Sections were blocked in 5% BSA/PBS (Sigma) and the primary antibodies were applied in 1% BSA/PBS at 4 °C overnight. The primary antibodies are listed in Table S3 and the verification of these antibodies is shown in Figure S5. Appropriate secondary antibodies (Zhongshan Goldenbridge Biotechnology, Co., Ltd., Beijing, China) were added and the slides were counterstained with Hoechst33342 (Sigma). For DAB immunohistochemistry, the HRP-conjugated secondary antibodies (Zhongshan Goldenbridge Biotechnology, Co., Ltd.) were used before DAB (Zhongshan Goldenbridge Biotechnology, Co., Ltd.) application. All images were obtained with a Nikon Fluorescence Microscope E80i (Nikon).

## 5. Conclusions

In conclusion, the present study highlights that during epidermal commitment, K8, p63 and K5/K14 exhibit a sequential, regular and highly position-specific expression pattern, which contributes to understanding the embryonic ectodermal development. Moreover, *Trp63* gene heterozygous mutations in humans are causative of some severe ectodermal dysplastic syndromes, which mainly occurs during embryonic development [41]. Uncovering the functions of p63 in epidermal development would be informative for the treatment of human ectodermal dysplastic syndromes.

## Supplementary Materials

Supplementary materials can be found at http://www.mdpi.com/1422-0067/16/12/26185/s1.
