# Supplementary material for: Spatiotemporal Expression of p63 in Mouse Epidermal Commitment
Source: Int J Mol Sci. 2015 Dec 10;16(12):29542–53. doi: 10.3390/ijms161226185 (PMC4691128; doi:10.3390/ijms161226185)
Supplement: Supplementary file 1 [file ijms-16-26185-s001.pdf]

Supplementary Materials: Spatiotemporal Expression of p63 in Mouse Epidermal Commitment

Qian Zhao, Shuang Liu, Huishan Zhang, Na Li, Xinyue Wang, Yujing Cao, Lina Ning, Enkui Duan and Guoliang Xia

Table S1. Antibodies used for whole-mount immunohistochemistry.

Primary Antibody	Vendor and Catalog	Dilution
p63	Santa Cruze, sc-8243	1:700
K8	Novus, NB 110-56919	1:1000
K5	Santa Cruze, sc-66836	1:500

Table S2. Primer sequences used for qRT-PCR and RT-PCR.

Gene	Primers
Trp63	Forward: AGACCACGCACAGAATAAGCG
	Reverse: TGGAAGGACACATCGAAGCTG
Krt8	Forward: CAAGGTGGAACTAGAGTCCCG
	Reverse: CTCGTACTGGGCACGAACTTC
Krt5	Forward: GGAGATCGCCACCTACAGGA
	Reverse: TCCGTAGCCAGAAGAGACACT
Gapdh	Forward: GTCCGTTGTGGATCTGACCT
	Reverse: CGGGAGATTCTCAGTGTGGT
TAp63	Forward: TGCATGCGGATACAATCC
	Reverse: GAGGAGCCGTTCTGAATCTG
ΔNp63	Forward: CAAAACCCTGGAAGCAGAAA
	Reverse: GAGGAGCCGTTCTGAATCTG

Table S3. Antibodies used for immunofluorescence staining and immunohistochemistry.

Primary Antibody	Vendor and Catalog	Dilution
p63	Santa Cruze, sc-8243	1:100
ΔNp63	Santa Cruze, sc-8431	1:100
K8	Novus, NB 110-56919	1:100
K14	Millipore, CBL197	1:100

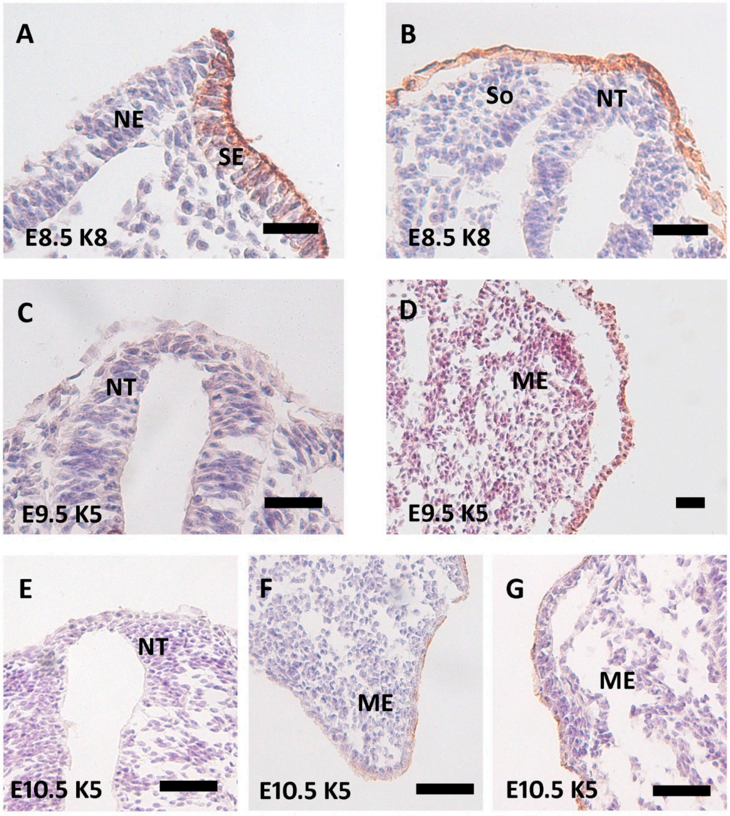

Figure S1. The sections of whole-mount immunohistochemistry embryos. (**A**,**B**) K8 expression in E8.5 embryos ((**A**) head field; (**B**) dorsal trunk containing a somite); (**C**,**D**) K5 expression in E9.5 embryos ((**C**) dorsal trunk containing the neural tube; (**D**) forelimb bud); (**E**–**G**) K5 expression in ((**E**) 10.5 embryos , neural tube; (**F**) forelimb bud; (**G**) posterior trunk containing a somite). NE, neural ectoderm; NT, neural tube; ME, mesenchyme; So, somite; SE, surface ectoderm. Bar = 50 μm.

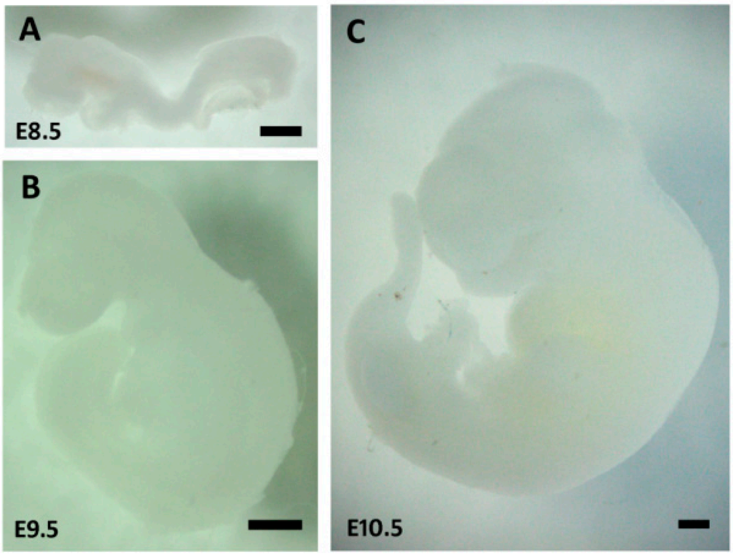

Figure S2. Negative controls for whole-mount immunohistochemistry embryos. (**A**) E8.5; (**B**) E9.5; (**C**) E10.5. Bar = 500 μm.

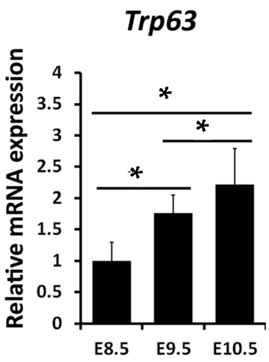

Figure S3. *Trp63* mRNA expression in E8.5–E10.5 embryos by qRT-PCR. * $p < 0.05$.

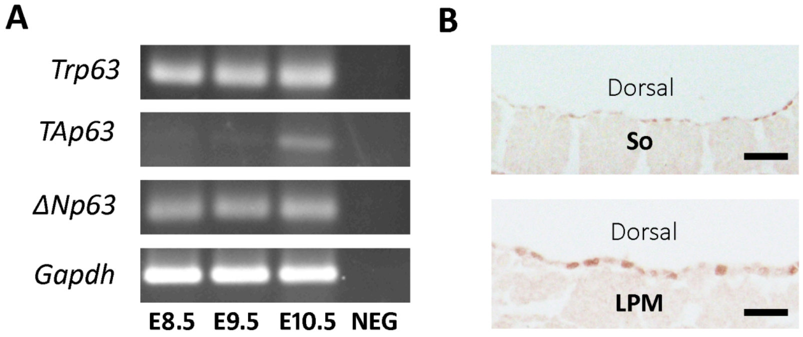

Figure S4. The expression of p63 isoforms in mouse embryos. (**A**) RT-PCR to examine the expression of *Trp63*, *TAp63* and *ΔNp6* in E8.5-E10.5 embryos; (**B**) ΔNp63 expression in surface ectoderm cells covering the newly formed somites (So) and lateral plate mesenchyme (LPM) at E8.5, NEG represented negative control. Bar = 50 μm.

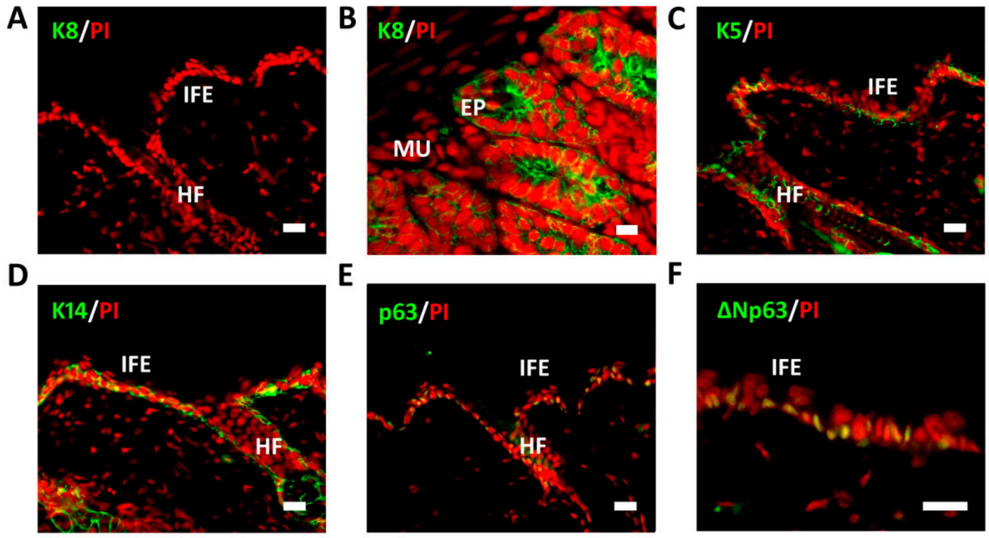

Figure S5. Verification of antibodies by immunofluorescence staining. (**A**) K8 (green) wasn't expressed in the mature mouse skin; (**B**) K8 (green) was specific expressed in the cytoplasm of mouse intestinal epithelial cells; (**C**,**D**) K5 (green) and K14 (green) were expressed in the cytoplasm of the basal layer cells in mature mouse skin; (**E**,**F**) p63 (green) and ΔNp63 (green) were expressed in the basal layer cell nuclei in mature mouse skin. PI was used to indicate the nuclei. HF, hair follicle; IFE, interfollicular epidermis; MU, mucosa; EP, epithelium. Bar = 20 μm.
